# Chemical Composition, Larvicidal and Ovicidal Activities, and Enzyme Inhibition Capacity of *Thymus serpyllum* Essential Oils Against *Spodoptera litura* (Fabricius)

**DOI:** 10.3390/plants13233315

**Published:** 2024-11-26

**Authors:** Lijun Wang, Siluo Jing, Shuang Wang, Zhikai Xing, Jiangyong Qu, Xumin Wang

**Affiliations:** College of Life Science, Yantai University, Yantai 264005, China; wanglijun@ytu.edu.cn (L.W.); jingsiluo@icloud.com (S.J.); wangshuang0456@126.com (S.W.); xingzhk@ytu.edu.cn (Z.X.)

**Keywords:** essential oil, enzyme inhibition, agricultural pest control, chemical composition

## Abstract

Due to their effectiveness at low doses and relative safety for non-target species, plant essential oils (EOs) are considered ideal alternatives to conventional pesticides for pest control. In this study, the chemical composition of *Thymus serpyllum* (*T. serpyllum*) EO was construed by Gas Chromatography-Mass Spectrometry (GC-MS), and its larvicidal and ovicidal activity against omnivorous pests *Spodoptera litura* (*S. litura*) was assessed. The effects of *T. serpyllum* EO on the activities of antioxidant detoxification enzymes were also measured. GC–MS analysis revealed that the main constituents of *T. serpyllum* EO were thymol (42.1%), p-cymene (22.4%), and γ-terpinene (18.6%). In the larvicidal toxicity experiment, the *T. serpyllum* EO demonstrated LC50 values of 0.606 and 0.664 mg/mL against the second- and third-instar larvae of *S. litura,* respectively, after 48 h exposure. Moreover, an EC_50_ value of 0.905 mg/mL was measured against *S. litura* eggs. In *S. litura*, *T. serpyllum* EO treatment reduced the enzymatic activity of ESTs and GST and, conversely, increased the enzymatic activity of AChE. Overall, this study demonstrated that *T. serpyllum* EO has the potential to be implemented as a novel eco-friendly insecticide against *S. litura*.

## 1. Introduction

*Spodoptera litura* (Fabricius, 1775) (Lepidoptera: Noctuidae) is a typical destructive crop pest worldwide [1]. Over 300 plant species have been identified as its hosts, including cotton, maize, vegetables, rice, groundnut, and soybean, strongly impacting the agricultural industry [2,3,4]. *S. litura* (*Spodoptera litura* Fabricius, 1775) (Lepidoptera: Noctuidae) exhibits a high reproductive and developmental ability, resulting in five to six overlapping generations annually. *S. litura* larvae are characterized by their ability to feed on leaves, buds, fruits, and flowers. If not treated in time, it might cause severe crop losses or even destruction [5,6]. Globally, synthetic insecticides are often used to control *S. litura*. However, due to their extensive application and due to long-term interactions between pesticides and insects, *S. litura* has developed resistance to many conventional pesticides, including organochlorides, organophosphates, cyantraniliprole, pyrethroids, abamectin, avermectins, and indoxacarb [1,7,8,9]. To date, severe insecticide resistance has been observed in *S. litura* in numerous countries, including China, Puerto Rico, Mexico, India, Pakistan, and Thailand [1,3].

In recent years, considering the non-selectivity and persistence of chemical pesticides, plant essential oils (EOs) and their derivatives garnered a growing interest due to their effectiveness at low doses [10] and relative safety to non-target organisms [11]. They can be completely degraded in the environment, leaving no residues, and are regarded as a valuable resource for the development and formulation of environmentally friendly pesticides with low toxicity. Recently, extracts or EOs from various plants such as *Crithmum maritimum* L. [12], *Couroupita guianensis* (Aubl.) [13], *Piper betle* L. [14], *Zanthoxylum armatum* DC. [15], *Inula racemosa* (Asteraceae) [16], *Zanthoxylum alatum* Roxb. [17], *Vernonia anthelmintica* L. [18], *Wedelia prostrata* (Hook. et Arn.) Hemsl. [19], *Acorus calamus* L. [20], *Alpinia galanga* (Linn.) Willd, and *Ocimum basilicum* L. [21] have been assessed on *S. litura* to examine their toxicity and antifeedant potential. Essential oils and their major constituents can serve as safe alternatives for pest control. Mahajan et al. [20] reported that β-caryophyllene exhibited a satisfactory inhibitory activity on the growth and development of *S. litura*. Yooboon et al. [21] discovered that piperine and β-asarone showed acute toxicity against *S. litura*, with their combination demonstrating greater acute toxicity than the individual compounds. Additionally, thymol [22], pogostone [23], and pulegone [3] were found to be effective against *S. litura*.

*Thyme* L. plants belong to the Lamiaceae family and are perennial herbs or low shrubs. More than 250 species of this genus have been recorded worldwide, with wide distribution in Northern Africa, temperate regions of Europe, and Asia [24]. Essential oils have been extensively adopted and utilized by the pharmaceuticals sector and the food sector for their potential biological properties and activities [25]. Previous studies have shown that *Thymus serpyllum* (L.) (*T. serpyllum)* EO has valuable biological activities, including antibacterial [26], antimicrobial [27], and antifungal activities [28]. Regarding its insecticidal activity, *T. serpyllum* EO exhibited notable efficacy in the control of *Frankliniella occidentalis* (Pergande) [29], *Acanthoscelides obtectus* (Say) (*A*. *obtectus*) [30], *Musca domestica* L. (*M. domestica*) [24], *Varroa destructor* (*V. destructor*) (Anderson and Trueman) [31] and *Reticulitermes dabieshanensis* (*R. dabieshanensis*) according to Wang et Li [32]. The composition of *T. serpyllum* EO has been inadequately studied [24,32], and its toxicity against *S. litura* remains unknown.

This study aimed to elucidate the insecticidal efficacy and mechanism of action of *T. serpyllum* essential oil against *S. litura*. In the present research, we hypothesized that it has considerable activity against *S. litura* larvae and eggs and can inhibit its growth by modulating enzyme activities. We investigated the effects of *T. serpyllum* EO on *S. litura* second- and third-instar larvae. The objectives of our study were to (1) analyze the composition of *T. serpyllum* EO using GC-MS; (2) evaluate the larvicidal and ovicidal activity of *T. serpyllum* EO on *S. litura*; and (3) explore the impact of *T. serpyllum* EO on *S. litura* detoxification enzymes. The findings will facilitate the formulation of efficient and low-toxicity botanical insecticides for the management of *S. litura.*

## 2. Results

### 2.1. Chemical Composition of T. serpyllum EO

The major constituents of the *T. serpyllum* EO are presented in Table 1. The *T. serpyllum* EO comprised 13 primary chemical constituents, accounting for 99.2% of its composition. The most abundant constituent was thymol (42.1%). Furthermore, other constituents included p-Cymene (22.4%), γ-Terpinene (18.6%), Carvacrol (3.6%), β-Pinene (2.7%), Linalool (2.5%), α-Pinene (1.6%), α-Terpinene (1.5%), Camphene (1.3%), Limonene (1.2%), Camphor (0.8%), Terpin-4-ol (0.6%), and α-Terpineol (0.3%).

### 2.2. Larval Toxicity

The toxicity of *T. serpyllum* EO to *S. litura* second- and third-instar larvae was evaluated using the leaf-dipping approach (Table 2 and Table 3). *T. serpyllum* EO toxicity varied in a dose-dependent manner. As shown in Table 2, the LC50 and LC90 reached 1.632 and 4.463 mg/mL in second-instar larvae after 12 h of exposure. After 24 h, the LC50 and LC90 reached 1.033 and 3.294 mg/mL, after 36 h, 0.780 and 2.317 mg/mL; after 48 h, 0.606 and 1.749 mg/mL; and after 60 h, 0.444 and 1.313 mg/mL. Following 72 h of exposure, LC50 and LC90 were 0.300 and 0.959 mg/mL, respectively. The larval toxicity of *T. serpyllum* EO against *S. litura* 2nd-instar larvae showed an increased trend with increased concentration. At the same time, the effective concentration showed a decreasing trend with more prolonged treatment and exposure time.

*T. serpyllum* EO demonstrated considerable efficacy against third-instar larvae of *S. litura*. As indicated in Table 3, the LC_50_ and LC_90_ values were 1.725 mg/mL and 4.401 mg/mL after 12 h of exposure. At a 0.25 mg/mL concentration, the mortality rate was 1.7 ± 2.9%, while it reached 63.3 ± 2.9% at 2.0 mg/mL. The toxicity of *T. serpyllum* EO to the third instar larvae increased with prolonged exposure. Mortality rates at each concentration rose progressively, with LC_50_ and LC_90_ values diminishing over time. After 72 h, they fell to 0.317 and 0.994 mg/mL, respectively. During this time point, the mortality rate reached 43.3 ± 10.4% at 0.25 mg/mL and increased to 100 ± 0.0% at 2.0 mg/mL. No mortality larvae mortality was observed in the control group treatment with acetone. One-way ANOVA analysis revealed a significant difference in mortality rates across the different EO concentrations (*p* < 0.0001). Tukey’s post hoc test also confirmed significant differences between the concentrations (*p* < 0.05). Logistic regression modelling indicated an EC50 value of 0.30 mg/mL at 72 h of exposure. These findings confirm that the toxicity of *T. serpyllum* EO to third-instar larvae of *S. litura* significantly increases with both exposure duration and concentration, while the effective concentration is reduced with increased exposure duration.

### 2.3. Ovicidal Activity

**Figure 1:**
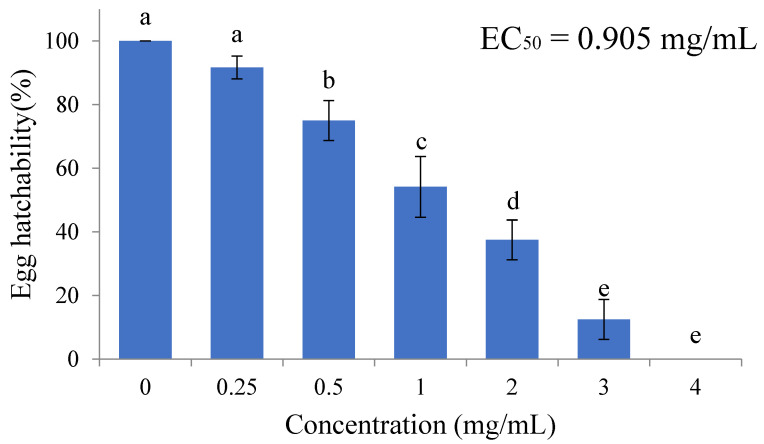
Ovicidal activity of the *Thymus serpillum* on eggs of *Spodoptera litura*. Values are mean ± SD of 3 replicates, and significant differences are indicated by different letters (a–e) (ANOVA, Tukey’s HSD, *p* < 0.05).

The ovicidal activity of *T. serpyllum* EO is shown in Figure 1. A dosage-dependent relationship was observed, with an EC_50_ = 0.905 mg/mL. The hatching rate in the 0, 0.25, 0.5, 1, 2, 3, and 4 mg/mL *T. serpyllum* EO treatment groups were 100 ± 0%, 91.7 ± 3.6%, 75.0 ± 6.3%, 54.2 ± 9.5%, 37.5 ± 6.3%, 12.5 ± 6.3%, and 0 ± 0%, respectively.

### 2.4. Activity Against S. litura Cellular Detoxification Enzymes

**Figure 2:**
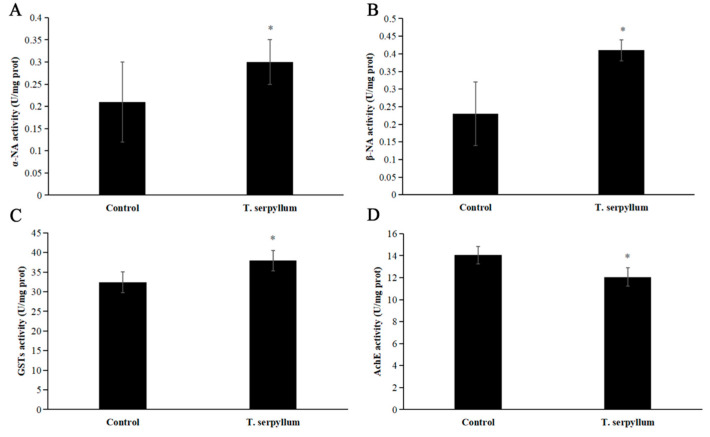
The effects of *Thymus serpillum* EO on the activities of detoxifying enzymes of *Spodoptera litura*. (**A**) Esterases (α-NA) activity for 24 h of LC_50_ treatment; (**B**) esterases (β-NA) activity for 24 h of LC_50_ treatment; (**C**) GSTs activity for 24 h of LC_50_ treatment; (**D**) AChE activity for 24 h of LC_50_ treatment. *p* < 0.05 were illustrated by the asterisk according to an independent sample *t*-test.

The potential role of *T. serpyllum* EO as an inhibitor of *S. litura* detoxifying enzymes involved in cellular detoxification was also investigated. CarE, GST, and AChE activities were measured, after 24 h of treatment with *T. serpyllum* (LC_50_ = 1.033 mg/mL). In the “Control group”, 10 μL of distilled water was used instead of 10 μL of the solution, and the rest of the reagents were kept unchanged. Among these three enzymes, AChE was significantly inhibited after treatment with *T. serpyllum* EO (Figure 2D). Compared with the control group, the α-NA activity, β-NA activity, and GST activities of *S. litura* were significantly increased by *T. serpyllum* EO (Figure 2A–C).

## 3. Discussion

The main constituent of *T. serpyllum* EO was thymol (42.1%), which is in accordance with the findings of Xie et al., Hýbl et al., and Yang et al. [24,31,32].

*S. litura* is a predominant polyphagousan pest [3] and has developed resistance to many chemical pesticides, such as benzoate, emamectin, carbamates, and pyrethroids [33]. Plant essential oils can serve as an alternative to biopesticides for pest control, targeting species of agricultural importance [17].

According to the study findings, the LC_50_ of *T. serpyllum* EO against *S. litura* 2nd, and 3rd instar larvae after a 48 h exposure was 0.606 mg/mL and 0.664 mg/mL, respectively. These findings suggest that *T. serpyllum* EO exhibits substantial toxicity towards *S. litura*. GC-MS analysis identified the primary constituents of *T. serpyllum* EO, namely thymol, carvacrol, (S)-(+)-carvone, estragole, citral, linalool, (S)-(-)-limonene, and γ-terpinene, all known for their insecticidal properties against larvae of various pests [33]. A limonene analogue resulted in morphological and physiological alterations in *Drosophila suzukii* L3 larvae, contributing to elevated larval mortality. This aligns with the findings by Yang [34], which reported that *T. serpyllum* EO exhibited high toxicity to *R. dabieshanensis*. Additionally, Hýbl et al. highlighted the insecticidal properties of *T. serpyllum* EO on *V. destructor* (LC50 = 2.549 μL/L) [31]. Xie et al. demonstrated that this essential oil also has toxic effects on *M. domestica* (LC50 = 20.9 μL/L) [24]. Sertkaya (2021) documented an LC_50_ of 1.12 μL/L for *T. serpyllum* EO against *A. obtectus*, along with a 90.0% repellent efficacy against *Frankliniella occidentalis* at a 0.5% concentration [35]. Similarly, *Lantana camara* EO exhibited potent insecticidal activity against *Aedes aegypti*, *Aedes albopictus*, and *Culex quinquefasciatus* larvae [36]. These findings align with prior research conducted by Yang et al. [32]. Similarly, *T. serpyllum* EO exhibited significant toxicity towards *R. dabieshanensis.* In addition, Hýbl et al. [31] demonstrated the insecticidal effect of *T. serpyllum* EO against *Varroa destructor* (LC_50_ = 2.549 μL/L). Xie et al. [24] showed *T. serpyllum* EO had toxic effects on *M. domestica* (LC_50_ = 20.9 μL/L). Research conducted by Sertkaya [30] indicated that the LC_50_ value of *T. serpyllum* EO against *A. obtectus* was 1.12 μL/L. Picard et al. [29] assessed the insecticidal ability of *T. serpyllum* EO, which showed a 90.0% repellent efficacy against *F*. *occidentalis* at 0.5%.

More recently, plant essential oils (EOs) have been implemented as a control agent against *S. litura* due to their effectiveness at low doses [10,11] and relative safety for non-target species [12]. Suresh et al. [10] reported that *C. maritimum* exhibited larvicidal activity against I-VI instar larvae of *S*. *litura* with LC_50_ values ranging from 102.1 to 237.0 μL/L. The larvicidal activity of a *C. guianensis* flower extract against *S. litura* third instar larvae (with an LC_50_ of 223 ppm) has been demonstrated by Ponsankar et al. [11]. The LC_50_ value of *Piper betle* L. EO against third instar *S. litura* larvae was 0.48%, as reported by Vasantha-Srinivasan et al. [12]. Similarly, Kaleeswaran et al. [13] reported that n-hexane (pericarp) *Z. armatum* pericarp extracts (0.209%), followed by Ethyl acetate (0.450%) and Methanol (0.654%) extracts, respectively, displayed larvicidal activity against third instar *S. litura* at 72 h. Benelli et al. [17] demonstrated that *W. prostrata* exhibited significant toxicity toward fourth instar larvae of *S. litura* (LC_50_ = 167.46 μL/mL). Strong toxicity of *A. galanga* (LD_50_ = 13.26 μg/larva) and *O. basilicum* (17.71 μg/larva) EO against *S. litura* has been reported by Ruttanaphan et al. [19].

The primary constituent of essential oils was identified as the determinant of their biological activity [24,31,34,35,36]. Thymol is one of the major volatile components of *T*. *serpyllum* EO. Ruttanaphan and Bullangpoti [3] demonstrated that thymol had significant toxicity against third instar larvae of *S. litura* after 24 and 48 h (LC_50_ = 5.610 μg/larva and 5.262 μg/larva). Koul et al. [22] reported a substantial insecticidal activity of thymol against *S. litura*, with LD_50_ of 28.5 μg/larva. Furthermore, thymol was determined to be toxic to various pest species (Table 4), including *R*. *dabieshanensis* [32,37,38]. As shown in Table 4, thymol has a broad-spectrum insecticide activity.

Previous studies have documented the toxicity of terpenoid complexes from essential oils to *S. litura* [3,18]. Mahajan et al. [18] determined the efficacy of β-caryophyllene, which resulted in a 13.33% adult emergence in *S. litura* at 3125 ppm. Similarly, β-asarone was toxic to *S. litura* larvae with LD_50_ values of 6.24 μg/larva [37]. Rotenone can be highly effective against third instar larvae of *S. litura* (LC_50_ = 5043 mg/L) [38]. According to Huang et al. [23], pogostone had significant larvicidal activity against *S. litura*, including oral toxicity (LC_50_ = 986.88 mg/L) and contact toxicity (LC_50_ = 1041.42 mg/L). Ruttanaphan and Bullangpoti [3] reported that pulegone exhibits high larvicidal activity against third instar larvae of *S. litura* after 24 h (LC_50_ = 8.348 μg/larva). Ruttanaphan et al. [21] discovered a remarkable insecticidal activity of linalool against *S. litura*, with an LD_50_ of 32.271 μg/larva and 49.742 μg/larva, respectively.

ESTs and GSTs are detoxification enzymes that play key roles in detoxifying botanical pesticides, and their activities are typically induced and up-regulated by exogenous compounds [84]. As shown in Figure 2, the ESTs and GSTs activities of *S. litura* were significantly elevated after exposure to an LC_50_ concentration of *T. serpyllum* EO. Yang et al. [32] reported that eight main components of EOs increased the activity of ESTs and GST of *R. dabieshanensis*; however, the activity of AChE was decreased. The EO from *C. citratus*, *C. khasans*, *C. nardus* and the main compound including citral, geraniol, and citronellal inhibited α-NA esterase activity and enhanced β-NA esterase activities [32]. The activities of ESTs and GST of *R. dabieshanensis* by treatment with M. citrate were significantly increased compared to the control [33]. This finding corresponded to studies by Yang et al. [32,84,85,86], which demonstrated the efficacy of *T. serpyllum* EO in enhancing the activity of detoxifying enzymes of treated insects.

Furthermore, AChE plays a significant role in the mechanism of action of essential oils (EOs) or relevant constituents, resulting in insecticidal effects. This study indicated that essential oil exerted a substantial inhibitory effect on acetylcholinesterase in vivo in *S. litura.* This is in agreement with findings by Jin et al. [34], who demonstrated that three Cymbopogon EOs and their primary components effectively inhibited the AChE activity of *R. ffaviceps* in vitro and in vivo. Additionally, Wu et al. [31] found that *Mentha* spp. EOs and their major constituents exhibited significant inhibitory activity against AChE both in vivo and in vitro. Previous studies have indicated that EOs and their major constituents can bind to the active site of AChE to inhibit AChE activity [34]. In addition, it has been demonstrated that most EOs can also exert toxic effects on insects by inhibiting cytochrome P450 enzymes (CYPs) [87], GABA receptors [88], octopus amine synapses [89], and tyramine receptors [90]. Therefore, EOs and their main constituents would lead to insect mortality by causing dysregulation in the nervous, antioxidant, and enzyme-based metabolic systems.

## 4. Materials and Methods

### 4.1. Insect Rearing

The eggs of *S. litura* were purchased from the Henan Jiyuan Baiyun Industry Company (Jiyuan, China). After the hatching, the larvae were raised individually on Chinese cabbage leaves. The laboratory temperature was maintained at 26 ± 2 °C, with 12:12 h (light/dark) cycles and RH of 75 ± 5%. The bioassay was conducted using eggs and healthy and uniform-sized 2nd and 3rd instar larvae.

### 4.2. Essential Oil

*T. serpyllum* EO was purchased from the online shop of Shanghai Zixin (Shanghai, China).

### 4.3. GC-MS Analysis

EOs of *T. serpyllum* were analyzed by an Agilent 7890B/5977A (Santa Clara, CA, USA) coupled with an HP-5 MS capillary column (30 m × 0.25 mm ID × 0.25 μm film thickness) (Santa Clara, CA, USA) and electron impact ionization (70 eV). Oven temperature increased at a rate of 10 °C min^−1^ from 50 °C (1 min) to 250 °C, subsequently kept at 250 °C for 2 min. Helium (99.999%) was applied as a carrier gas. Through GC-MS analysis of the essential oils, the peak areas of the major constituents were recorded in this study, and the relative percentage of each component was calculated by peak area normalization ($P_{i}$):

$$P_{i}=\frac{A_{i}}{A_{total}}\times 100\%$$

where *i* is the individual compound;$\;A_{i}$ is the peak area per compound; and $A_{total}$: is the sum of all compound peak areas corresponding to the total peak area.

The peaks of EOs of *T. serpyllum* were annotated by comparing the retention indices (RI) and mass spectra (NIST 11.0) and Wiley 275 library data with those of the literature.

### 4.4. Larvicidal Activity

Larvicidal bioassays were carried out using the modified leaf dipping method as described by Benelli et al. [17]. The *T. serpyllum* EO was evaluated in a series of concentration gradients (0, 0.25, 0.5, 1.0, and 2.0 mg/mL) diluted in the solvent Polysorbate 80 (P8010, Sigma-Aldrich, St. Louis, MO, USA). Then, Chinese cabbage leaves of uniform shape and size (3 cm × 3 cm) were immersed in the series of concentration gradients separately. After 10 s, the leaf discs were removed and dried, then placed in moisturized filter paper on plates. After that, each of the 20 larvae was placed onto Chinese cabbage leaves treated with different EO concentrations, respectively. The number of dead larvae was counted every 12 h and observed for 72 h. All experiments were performed in triplicate.

### 4.5. Ovicidal Bioassays

A total of 420 eggs were split into 7 groups (*n* = 60 eggs in each group). Each group was separately immersed in different concentrations (0, 0.25, 0.5, 1.0, and 2.0 mg/mL in Polysorbate 80) of *T. serpyllum* EO. Then, the number of eggs hatched in each group was counted. All experiments were performed in triplicate. Hatching rates were assessed at 120 h after the initiation of the treatment.

### 4.6. Enzyme Activities

#### 4.6.1. Homogenate Preparation

The 2nd instar larvae were placed into the LC_50_ concentrations of *T. serpyllum* EO to evaluate the impact on detoxification enzymes, specifically esterases and acetylcholinesterase. Each group (20 larvae) was homogenized, and centrifuged at 12,000× *g* for 15 min at 4 °C. Then, the supernatants were stored at −80 °C for subsequent use. Each assay was repeated a minimum of three times.

#### 4.6.2. Esterases (ESTs)

The EST assay was carried out based on the protocol of Piri et al. [56]. The EST was determined on two substrates, acetate-1-naphthyl ester (α-NA) (N1252, Sigma-Aldrich, St. Louis, MO, USA), and acetate-2-naphthyl ester (β-NA) (N1254, Sigma-Aldrich, St. Louis, MO, USA). The 20 µL substrate (α-NA or β-NA) (10 mM) and 50 µL Fast Blue RR Salt (1 mM) (F6250, Sigma-Aldrich, St. Louis, MO, USA) were mixed. Finally, 10 μL of the enzyme solution was supplemented into the mixture. In the control group, the 10 μL enzyme solution was replaced by 10 μL distilled water, and the rest of the reagents remained unchanged. After incubation at 27 °C for 5 min, the absorbance (OD value) was determined at 450 nm using a microplate reader (Synergy™ HTX, BioTek, Winooski, VT, USA). In addition, OD values were recorded every 1 min for 10 min. Each assay was repeated at least three times to ensure the reliability and reproducibility of the data. The EST activity was calculated using the following formula:

EST activity (U/mg) = (ΔA_test_ − ΔA_ck_) × V_total_ ÷ V_test_ ÷ T

ΔA_test_ corresponds to the absorbance change value of the treatment group measured for 10 min. ΔA_ck_ corresponds to the change in absorbance of the control group measured for 10 min. V_total_ represents the total volume of the reaction system in each well. V_test_ represents the volume of the enzyme solution. T denotes the reaction time.

#### 4.6.3. Glutathione S-Transferases (GSTs) Activity Assay

The enzymatic activity of GSTs was measured using the protocol employed by Piri et al. [56]. 20 μL CDNB (20 mM) and 510 μL enzyme solution were mixed. After incubation at 27 °C for 5 min, the absorbance was determined at 340 nm. Each assay was repeated a minimum of three times. The GST activity was calculated using the formula below:

GST activity (U/mg) = (Δ_OD340_ × V_total_) ÷ (ε × L)

Δ_OD340_ corresponds to the GST-mediated absorbance change. V_total_ represents the total volume of the reaction system in each well. ε represents the molar extinction coefficient of GST (0.0096/(μmol·cm)), and L represents the optical range of the colourimetric cup (1 cm).

#### 4.6.4. Acetylcholinesterase (AChE) Activity Assay

AChE (C4359, Sigma-Aldrich, St. Louis, MO, USA) activity was determined following the protocol of Ellman et al. [91]. 80 μL PBS solution (0.1 M, pH = 7.0), 50 μL ATCh (10 mM), and 50 μL 5,5-dithiobis-2-nitrobenzoic acid (DTNB) (D8130, Sigma-Aldrich, St. Louis, MO, USA) (10 mM) were mixed, respectively. After incubation at 27 °C for 5 min, 20 μL of enzyme solution was then added. Absorbance (OD) was measured at 405 nm using a microplate reader, and OD values were recorded at 1 min intervals for 30 min. Each assay was repeated at least three times to ensure the reliability and reproducibility of the data. The AChE activity was calculated using the following formula:

AChE activity (U/mg) = (Δ_OD405_ × V_total_) ÷ (ε × L)

Δ_OD405_ corresponds to AChE-mediated absorbance change. V_total_ represents the total volume of the reaction system in each well. ε represents the molar extinction coefficient of AChE (0.0136/(μmol·cm)), and L represents the optical range of the colourimetric cup (1 cm).

### 4.7. Statistical Analysis

Larval mortality, hatching, and suppression rates were analyzed using nonparametric statistical methods. The Kruskal–Wallis test was implemented to evaluate overall differences among treatment groups (*p* < 0.05). Dunn’s post hoc test was used to identify specific groups with significant differences in pairwise comparisons. All statistical analyses were performed with SPSS v20.0 (SPSS Inc., Chicago, IL, USA). The LC_50_ and EC_50_ values were calculated by fitting dose–response curves with logistic regression models. Within-group differences were assessed using the independent samples Mann–Whitney U-test, denoting results with *p* < 0.05 with an asterisk (*).

## 5. Conclusions

In our study, thymol was the main constituent of *T. serpyllum* EO. Moreover, *T. serpyllum* EO was highly toxic to second- and third-instar larvae of *S. litura*. Moreover, it demonstrated significant inhibition of AChE activity, confirming that *T. serpyllum* EO can be developed and utilized control agent against *S. litura*. Before large-scale field application, the effects of *T. serpyllum* EO on non-target organisms must be determined. There is also a need to design slow-release formulations that can be used to extend the effectiveness of *T. serpyllum* EO. The findings disclosed in this study may facilitate the effective and environmentally friendly management of *S. litura* in the field.

## Figures and Tables

**Table 1:** Components of *Thymus serpillum* by GC-MS.

No	Compounds ^a^	RI ^b^	RI ^c^	(%)
1	α-Pinene	937	936	1.6
2	Camphene	954	954	1.3
3	β-Pinene	977	979	2.7
4	α-Terpinene	1018	1017	1.5
5	p-Cymene	1025	1025	22.4
6	Limonene	1030	1029	1.2
7	γ-Terpinene	1060	1060	18.6
8	Linalool	1099	1097	2.5
9	Camphor	1146	1134	0.8
10	Terpin-4-ol	1180	1177	0.6
11	α-Terpineol	1191	1186	0.3
12	Thymol	1292	1290	42.1
13	Carvacrol	1307	1299	3.6

^a^ Components in order of elution from an HP-5 MS column. ^b^ RI, retention index computed on the HP-5MS column relative to C8–C28 n-alkanes. ^c^ Relative retention indices taken from Adams.

**Table 2:** Second-instar larvael activity of the *Thymus serpillum* EO.

Time (h)	Concentration (mg/mL)	Mortality (%) ± SD	LC_50_ (mg/mL) (95%CL *)	LC_90_ (mg/mL) (95%CL)	χ2
12	0.25	3.3 ± 2.9 b	1.632 (1.281–2.378)	4.463 (2.886–11.051)	15.980
	0.5	5.0 ± 5.0 b			
	1.0	18.3 ± 5.8 b			
	2.0	66.7 ± 10.4 a			
		F_3,8_ = 60.317, *p* < 0.0001			
24	0.25	11.7 ± 2.9 c	1.033 (0.815–1.393)	3.294 (2.186–7.114)	15.789
	0.5	16.7 ± 2.9 c			
	1.0	35.0 ± 5.0 b			
	2.0	83.3 ± 10.4 a			
		F_3,8_ = 85.481, *p* < 0.0001			
36	0.25	15.0 ± 0.0 c	0.780 (0.667–0.919)	2.317 (1.794–3.384)	10.300
	0.5	23.3 ± 7.6 c			
	1.0	53.3 ± 7.6 b			
	2.0	93.3 ± 2.9 a			
		F_3,8_ = 120.600, *p* < 0.0001			
48	0.25	20.0 ± 5.0 c	0.606 (0.517–0.707)	1.749 (1.386–2.460)	10.254
	0.5	35.0 ± 8.7 c			
	1.0	65.0 ± 5.0 b			
	2.0	98.3 ± 2.9 a			
		F_3,8_ = 108.062, *p* < 0.0001			
60	0.25	31.7 ± 2.9 c	0.444 (0.369–0.520)	1.313 (1.049–1.835)	8.827
	0.5	46.7 ± 10.0 c			
	1.0	80.0 ± 5.0 b			
	2.0	100 ± 0.0 a			
		F_3,8_ = 81.784, *p* < 0.0001			
72	0.25	46.7 ± 2.9 b	0.300 (0.228–0.365)	0.959 (0.767–1.357)	9.744
	0.5	65.0 ± 10.0 b			
	1.0	90.0 ± 10.0 a			
	2.0	100 ± 0.0 a			
		F_3,8_ = 33.640, *p* < 0.0001			

CL *: confidence limit which has been calculated with 95% confidence. The symbols “a”, “b” and “c” in the table denote levels of statistical significance, indicating meaningful differences between groups.

**Table 3:** Third-instar Larval activity of the *Thymus serpillum* EO.

Time (h)	Concentration (mg/mL)	Mortality (%) ± SD	LC_50_ (mg/mL) (95%CL)	LC_90_ (mg/mL) (95%CL)	χ2
12	0.25	1.7 ± 2.9 c	1.725 (1.458–2.174)	4.401 (3.205–7.486)	13.964
	0.5	5.0 ± 0.0 c			
	1.0	15.0 ± 5.0 b			
	2.0	63.3 ± 2.9 a			
		F_3,8_ = 235.933, *p* < 0.0001			
	0.25	11.7 ± 2.9 c	1.159 (0.962–1.462)	4.218 (2.918–7.646)	9.307
	0.5	15.0 ± 5.0 c			
	1.0	35.0 ± 5.0 b			
	2.0	78.3 ± 2.9 a			
		F_3,8_ = 169.333, *p* < 0.0001			
36	0.25	13.3 ± 2.9 d	0.818 (0.696–0.972)	2.554 (1.945–3.843)	4.581
	0.5	23.3 ± 2.9 c			
	1.0	55.0 ± 5.0 b			
	2.0	88.3 ± 2.9 a			
		F_3,8_ = 276.000, *p* < 0.0001			
48	0.25	18.3 ± 2.9 d	0.664 (0.560–0.786)	2.166 (1.657–3.244)	5.220
	0.5	35.0 ± 5.0 c			
	1.0	60.0 ± 5.0 b			
	2.0	93.3 ± 2.9 a			
		F_3,8_ = 191.667, *p* < 0.0001			
60	0.25	31.7 ± 2.9 c	0.467 (0.389–0.547)	1.398 (1.112–1.967)	8.629
	0.5	45.0 ± 5.0 c			
	1.0	76.7 ± 5.8 b			
	2.0	100 ± 0.0 a			
		F_3,8_ = 142.500, *p* < 0.0001			
72	0.25	43.3 ± 10.4 c	0.317 (0.245–0.382)	0.994 (0.796–1.399)	6.136
	0.5	65.0 ± 5.0 b			
	1.0	88.3 ± 5.8 a			
	2.0	100 ± 0.0 a			
		F_3,8_ = 45.667, *p* < 0.0001			

The symbols “a”, “b” and “c” in the table denote levels of statistical significance, indicating meaningful differences between groups.

**Table 4:** Insecticidal activity of *Thymus serpyllum* EO and its major component thymol.

Oil/Compounds	Insect Species	LC50 or LD_50_	References
*Thymus serpyllum* EO	*Reticulitermes dabieshanensis*	0.092 μL/L	Yang et al. [32]
	*Varroa destructor*	2.549 μL/L	Hýbl et al. [31]
	*Musca domestica*	20.9 μL/L	Xie et al. [24]
	*Acanthoscelides obtectus*	1.12 μg/mL	Sertkaya [30]
	*Frankliniella occidentalis*	0.5%	Picard et al. [29]
thymol	*Amblyomma sculptum;*	0.0156 mg/cm^2^	da Silva Costa et al. [39]
	*Rhipicephalus sanguineus*	0.0041 mg/cm^2^	
	*Aedes aegypti*	0.1 mg/mL 100% mortality	Nascimento et al. [40]
	*Reticulitermes dabieshanensis*	0.062 μL/L	Yang et al. [32]
	*Tribolium castaneum;*	24.65 μg/adult;	Xie et al. [41]
	*Lasioderma serricorne;*	9.9 μg/adult;	
	*Liposcelis bostrychophila*	49.36 μg/adult	
	*Solenopsis invicta*	0.98 μg/g, minimum repellent effective doses	Paudel et al. [42]
	*Acromyrmex balzani*	2.23 µg/mg	Dantas et al. [43]
	*Chilo suppressalis*	17.11 μg/larvae	Basij et al. [44]
	*Plutella xylostella*	2.45 mg/mL	Zhao et al. [45]
	*Sitophylus oryzae*	51.84%, repellency effects for 2%	Marsin and Muhamad [46]
	*Tribolium confusum;*	0.3%	Amari et al. [47]
	*Supella longipalpa*		
	*Sitophilus zeamais*	196 μmol/cm^2^	Rodríguez et al. [48]
	*Glyphodes pyloalis*	32.18 μg/larva	Goharrostami et al. [49]
	*Spodoptera exigua*	9.54 μg/larva	Kumrungsee et al. [50]
	*Spodoptera litura*	5.610 μg/larva	Ruttanaphan and Bullangpoti [3]
	*Acanthoscelides obtectus*	Females, 98.4 mg/kg;	Lazarević et al. [51]
		Males, 66.0 mg/kg	
	*Plutella xylostella*	27.94 mg/L	da Camara et al. [52]
	*Spodoptera exigua*	32.45 μg/larva	Pengsook et al. [53]
	*Galleria mellonella*	0.5 mg/adult	Sohail et al. [54]
	*Podisus nigrispinu;*	10.27 mg/g;	Lima et al. [55]
	*Spodoptera frugiperda*	4.91 mg/g	
	*Tuta absoluta*	7.72 μL/mL	Piri et al. [56]
	*Mythimna separate* *;*	6.67 μL/L;	Lu et al. [57]
	*Myzus persicae* *;*	5.58 μL/L;	
	*Sitophilus zeamais* *;*	59.20 μL/L;	
	*Musca domestica* *;*	1.66 μL/L;	
	*Tetranychus cinnabarinus*	2.14 μL/L	
	*Riptortus clavatus*	70.0% repellent activity at 2.83 μg/cm^2^	Lee et al. [58]
	*Culex pipiens*	49 mg/L	Youssefi et al. [59]
	*Leishmania infantum*	7.22 μg/mL	Youssefi et al. [60]
	*Musca domestica*	13 mg/L	Scalerandi et al. [61]
	*Sitophilus zeamais*	84.06 μL/L	Oliveira et al. [62]
	*Aedes albopictus*	12.9 mg/L	Giatropoulos et al. [63]
	*Hyalomma lusitanicum*	100% at 5 mg/L	Navarro-Rocha et al. [64]
	*Plutella xylostella*	0.00018 ppm	Webster et al. [65]
	*Aedes aegypti*	35.71 ppm	de Mesquita et al. [66]
	*Diaphania hyalinata*	2.99 μg/mg	Melo et al. [67]
	*Blatta lateralis*	0.34 mg/nymph	Gaire et al. [68]
	*Sitophilus zeamais*	17.08 μg/mg	Oliveira et al. [69]
	*Cryptotermes brevis*	8.20 μg/mg	Santos et al. [70]
	*Ixodes ricinus*	100% at 1%	Tabari et al. [71]
	*Aedes aegypti*	11.1 μg/cm^2^	Ali et al. [72]
	*Blattella germanica*	100%, repellency effects for 10 μg/cm^2^	Lee et al. [73]
	*Sitophilus oryzae* *;*	24.07 μg/cm^2^;	Kanda et al. [74]
	*Tribolium castaneum;*	11.21 μg/cm^2^	
	*Rhyzopertha dominica;*	8.8 μg/cm^2^	
	*Aedes aegypti*	0.013 mg/cm^2^	Rehman et al. [75]
	*Aedes albopictus*	100%, larvicidal activity, 0.1 mg/mL	Seo et al. [76]
	*Stegomyia aegypti*	68.05 μg/cm^2^	Huang et al. [77]
	*Plutella xylostella*	0.22 μg/larva	Kumrungsee et al. [78]
	*Aedes aegypti*	13.9 ppm	Tabanca et al. [79]
	*Spodoptera litura*	28.5 μg/larva	Koul et al. [22]
	*Reticulitermes speratus*	0.65 mg/Petri dish	Sekine and Shibutani [80]
	*Crithidia fasciculata;*	32.5 μg/mL;	Azeredo and Soares [81]
	*Trypanosoma cruzi*	62 μg/mL	
	*Aedes albopictus*	9 μL/L	Park et al. [82]
	*Tenebrio molitor*	14.71 μL/L	Lima et al. [83]

## Data Availability

No new data were created or analyzed in this study. Data sharing is not applicable to this article.
